# Two patients with intestinal failure requiring home parenteral nutrition, a *NOD2* mutation and tuberculous lymphadenitis

**DOI:** 10.1186/1471-230X-14-43

**Published:** 2014-03-06

**Authors:** Holger Schäffler, Matthias Teufel, Sabrina Fleischer, Chih-Jen Hsieh, Julia-Stefanie Frick, Georg Lamprecht

**Affiliations:** 1 Division of Gastroenterology, Department of Medicine II, University of Rostock, Ernst-Heydemann-Str. 6, D-18057 Rostock, Germany; 2 Department of Diagnostic Radiology, Eberhard Karls University Tübingen, Tübingen, Germany; 3 1st Medical Department, University of Tübingen, Tübingen, Germany; 4 Institute of Medical Microbiology and Hygiene, University of Tübingen, Tübingen, Germany

**Keywords:** NOD2, Intestinal failure, Tuberculous lymphadenitis, Catheter related blood stream infection

## Abstract

**Background:**

Mutations in the *NOD2* gene are a significant risk factor to acquire intestinal failure requiring home parenteral nutrition. Tuberculous lymphadenitis is the main manifestation of extrapulmonary tuberculosis. Defects in the innate immunity, including *NOD2* mutations, may increase the risk for acquiring infections caused by *M. tuberculosis*. An association of intestinal failure, mutations in the *NOD2* gene and tuberculous lymphadenitis has not been described before.

**Case presentation:**

We report of two patients with intestinal failure secondary to mesenteric ischemia. Both patients presented with fever and weight loss while receiving long term home parenteral nutrition. Both of them were found to have mutations in the *NOD2* gene. Catheter related infections were ruled out. FDG-PET-CT scans initially obtained in search for another infectious focus that would explain the symptoms unexpectedly showed high FDG uptake in mediastinal lymph nodes. Direct or indirect evidence proved or was highly suggestive for tuberculous lymphadenitis. Intravenous tuberculostatic therapy was started and led to a reversal of symptoms and to resolution of the lesions by FDG-PET-CT.

**Conclusion:**

Mutations in the *NOD2* gene may put patients both at an increased risk for acquiring *M. tuberculosis* infections as well as at an increased risk of intestinal failure after extensive intestinal resection. Thus we suggest to specifically include reactivated and opportunistic infections in the differential diagnosis of suspected catheter related infection in patients with intestinal failure who carry mutations in their *NOD2* gene.

## Background

Tuberculous lymphadenitis is the most frequent site of extrapulmonary tuberculosis. About 20% of all TBC cases in the US are extrapulmonary. From this group, about 40% are tuberculous lymphadentis [1]. Detection of *M. tuberculosis* is mainly via the innate immune system by extracellular or intracellular pattern recognition receptors (PRR) such as toll-like receptors (TLR) and nucleotide-binding oligomerization domain receptors (NOD) [2-4]. Mutations in specific TLR genes were found to be associated with susceptibility to TBC [5,6].

Clinical relevance of mutations in the *NOD2* gene arises from their association with Crohn’s disease [7-10]. In 2001, a link between mutations in the *NOD2* gene and Crohn’s disease was first established independently by two different groups [11,12]. However, the exact function of *NOD2* is still under debate [13]. Besides Crohn’s disease, other disease entities seem to be related to mutations in the *NOD2* gene like GvHD [14-16], acute septicemia [17], spontaneous bacterial peritonitis in liver cirrhosis [18,19] and worsened outcome after intestinal transplantation [20]. Mutations in the *NOD2* gene and an increased susceptibility for infectious diseases have been reported in the literature [21]. *NOD2* is also thought to be an important receptor in recognizing *M. tuberculosis*, because on the one hand both the receptor and the pathogen are intracellular and on the other hand the cell wall of *M. tuberculosis* contains peptidoglycan, which is one of the ligands of *NOD2*[22]. However, the role of *NOD2* in tuberculous lymphadenitis has not been studied yet. To this end a recent study has described a new SNP in the *NOD2* gene as a possible risk factor for pulmonary tuberculosis in the Chinese Han population [23]. In another study it was reported that genes in the *NOD2* signaling pathway are associated with susceptibility to infections with *Mycobacterium leprae* in China [24].

Short bowel syndrome (SBS) and intestinal failure requiring long term home parenteral nutrition (HPN) are rare heterogeneous clinical conditions in which extensive parts of the intestine have been removed surgically. The main causes of short bowel syndrome in adults are Crohn’s disease, intestinal ischemia, volvulus, ileus, desmoid tumors and trauma [25]. Recently we have described an increased frequency of *NOD2* mutations in SBS patients without underlying Crohn’s disease [26]. Infections associated with intestinal failure requiring home parenteral nutrition are mainly catheter-related [27,28].

Here we describe two individuals with short bowel syndrome, mutations in the *NOD2* gene and tuberculous lymphadenitis. This is a clinically important finding because in a HPN patient intermittent fever, the key symptom of tuberculous lymphadenitis, usually indicates catheter related blood stream infection. To our best knowledge, this is the first case report linking these clinical entities together.

## Materials and methods

Genotyping of patients was performed as part of a larger study in a cohort of short bowel patients, which was approved by the Ethics Committee of the University of Tuebingen (022/2011BO2). The patients gave written informed consent. The three major mutations in the *NOD2* gene (SNP 8; R702W, NCBI reference SNP ID: rs2066844 and SNP 12; G908R, NCBI reference SNP ID: rs2066845 and 3020insC, SNP 13; 1007 fs, NCBI reference SNP ID: rs2066847) were detected in genomic DNA extracted from whole blood as described previously [26].

Direct Nucleic Acid Amplification Test (NAAT) to detect *M. tuberculosis* complex DNA was performed using the ProbeTec ET DTB (DTB) (Becton-Dickinson). For identification of *Mycobacterium tuberculosis* complex species GenoType® MTBC (Hain) was used according to manufacturer’s instructions. As gold standard culture techniques using 2 solid and BACTEC™ *MGIT*™ 960 liquid broth were applied. For direct susceptibility testing we used the BACTEC™ *MGIT*™ 960. As interferon-gamma-release assay we used the *QuantiFERON*-TB® Gold In-Tube test (Cellestis) according to manufacturer’s instructions.

## Case presentation

### Patient 1

**Figure 1:**
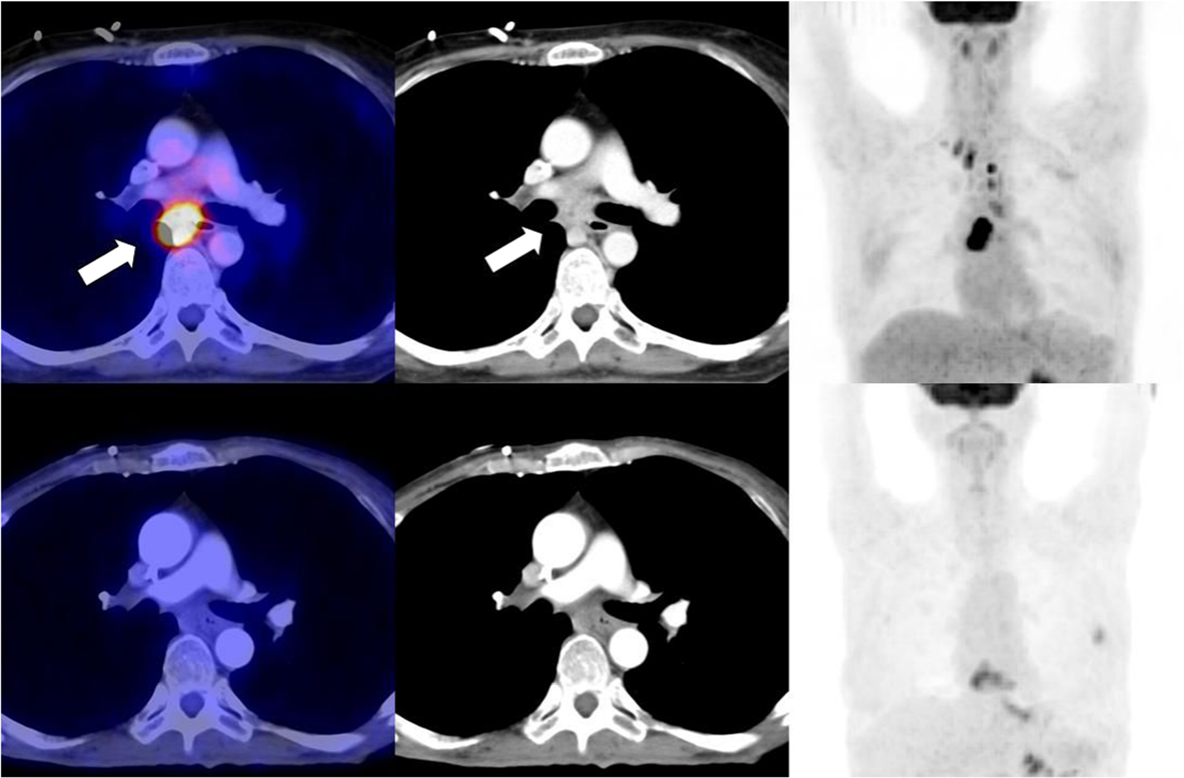
**Patient 1.** Upper row - before tuberculostatic therapy: High FDG uptake in an infracarinal lymph node (left panel: fusion images of PET/CT) correlating with central necrosis in the contrast enhanced CT (middle panel). Coronal MIP with high FDG uptake in several mediastinal lymph nodes (right panel). Lower row - 8 month follow up: No FDG uptake (left panel: PET/CT) and significant size reduction of the visualized lymph nodes (middle panel: ceCT). No pathological FDG uptake in the mediastinal lymph nodes on coronal MIP of PET (right panel).

Patient 1 is a 44 year-old Caucasian woman with intestinal failure. In 2008, she required surgical resection of most of her small intestine (except for 70 cm of proximal jejunum) and the right colon resulting in a duodenotransversostomy due to acute occlusion of her superior mesenteric artery. Total parenteral nutrition was initiated at the University of Tübingen intestinal failure outpatient clinic. The initial clinical course was dominated by numerous infectious complications, e.g. recurrent line infections (05/2009, 04/2010, 02/2012), a liver abscess and an episode of acute cholecystitis in the absence of cholelithiasis, which was interpreted as another ischemic episode in the splanchnic circulation (Figure 1).

Despite an extensive workup (including ultrasound and CT-scan) the etiology of the ischemic events could not be determined. The diagnosis of Takayasu Arteriitis was entertained because of diminished/absent peripheral pulses but vasculitis was neither found by histology in the resected specimens nor by PET-CT in the large vessels. Workup for a coagulation disorder revealed a heterozygous prothrombin mutation (G20210A). An antiphospholipid syndrome was ruled out and the JAK2-mutation was also not detected. A HIV test was negative.

Because of the severity of two acute episodes of arterial occlusion the patient was maintained on low dose steroids (Prednisolone 5 mg) and received long term anticoagulation with enoxaparin (Clexane) and later fondaparinux (Arixtra). Under this regimen, the patient did not develop another episode of intestinal or systemic arterial ischemia.

Genetically she was found to be heterozygous for the 1007 fs *NOD2* mutation. In 2010, after two years of successful parenteral nutrition without any infectious complications the patient started to lose weight and had intermittent episodes of fever. The laboratory values showed an increased CRP value (30.5 mg/dl) and anemia (6.7 g/dl). A catheter-related infection was excluded by repeated blood cultures. Another FDG-PET-CT was obtained addressing again the question of a large vessel vasculitis. Unexpectedly it showed for the first time an intensive uptake of the FDG tracer in three lymph nodes in the mediastinum. An interferon-γ-release assay was positive (Quantiferon®). A transbronchial biopsy of one of the lymph nodes revealed necrotic material and a granuloma by histology, highly suggestive for tuberculous lymphadenitis. *M. tuberculosis-*DNA was detected by PCR and cultures obtained from the lymph node also grew *M. tuberculosis*. Intravenous tuberculostatic therapy with Ethambutol, Isoniazid and Rifampicin was given for 4 months followed by Isoniazid and Rifampicin for another 8 months. The follow-up FDG-PET-CT after 7 months of therapy showed a significant size reduction and decreased FDG uptake of the lymph nodes in the mediastinum. The patient stayed on prophylactic therapy with Isoniazid until today, because she continued low dose steroids.

### Patient 2

**Figure 2:**
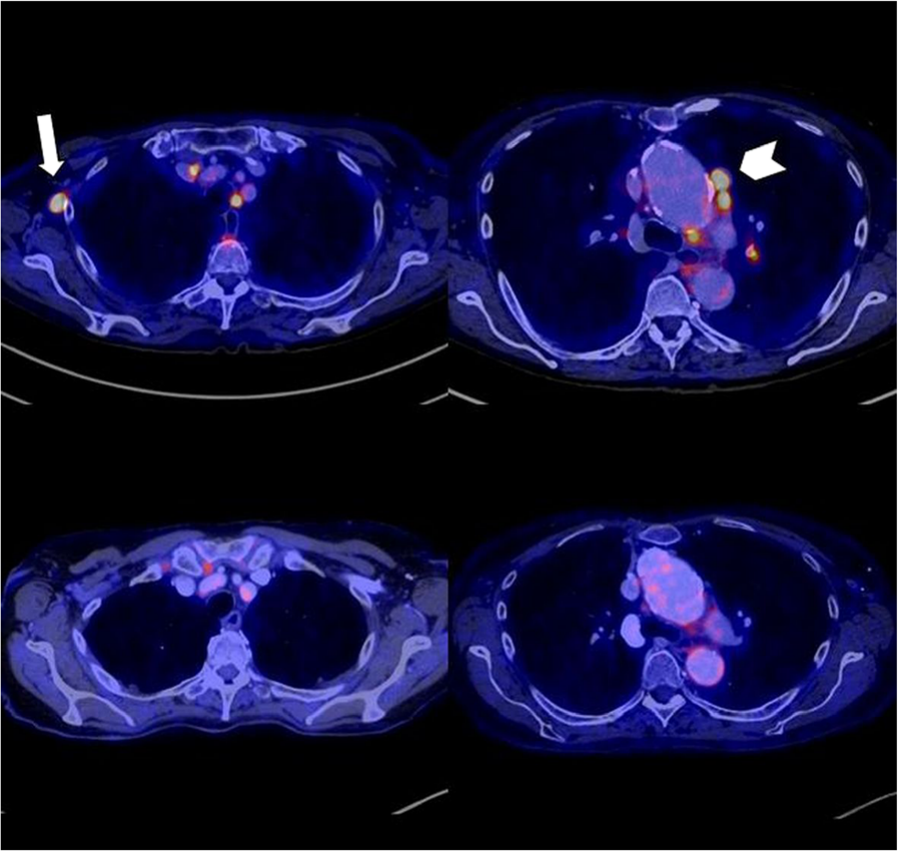
**Patient 2.** Upper row - before tuberculostatic therapy: high FDG uptake in several mediastinal and one right axillary (arrow) lymph nodes. Additionally, further lymph nodes with high FDG uptake, especially in the left paraaortal (arrow head) region. Lower row - 7 month follow up after initiation of tuberculostatic therapy: No FDG uptake in the right axillary lymph nodes and significant regression of FDG uptake of the paraaortal lymph nodes after therapy. All PET positive lymph nodes had a morphological correlate and showed a significant size reduction after therapy in contrast enhanced CT (not shown).

Patient 2 is a 77 year-old Caucasian woman who had an infarction of the small intestine and right colon due to atherosclerotic occlusion of the superior mesenteric artery in November 2003. She required extensive resection of her small bowel, which resulted in a jejunotransversostomy with 20 cm of proximal jejunum. Total parenteral nutrition was started and was managed at the University of Tübingen intestinal failure outpatient clinic. In 2010 she developed fever, weight loss and night sweats. An elevated ESR and LDH were found. Repeated attempts to verify catheter related blood stream infection including numerous blood cultures, several rounds of empiric antibiotic therapy and an empiric exchange of the catheter had no sustained effect on these symptoms. A FDG-PET-CT scan was performed addressing a potential infectious focus other than the catheter. Unexpectedly it revealed PET-positive lymphadenopathy in the cervical region and in the mediastinum. An interferon-γ-release assay was negative (Quantiferon®). A bronchoscopic biopsy of the suspicious lymph node revealed granulomatous necrotizing lymphadenitis, but acid fast bacilli could not be stained. A specific pathogen could not be cultured and eubacterial PCR as well as PCR for *M. tuberculosis* were negative. The family history revealed that several relatives had suffered from tuberculosis. Based on the sum of indirect evidence the diagnosis of tuberculous lymphadenitis was made. Tuberculostatic therapy (Isoniazid, Rifampicin, Ethambutol and Levofloxacin) was applied intravenously for 6 months. The patient soon felt better and gained weight. Fever and anemia resolved, and the LDH and the ESR returned to normal values. Regression of the enlarged and hypermetabolic lymph nodes was verified by another PET-CT-scan obtained 7 months after the initiation of specific therapy. One remaining small mediastinal lymph node was regarded as non-specific, (Figure 2).

The patient was found to be heterozygous for the R702W mutation in the *NOD2* gene.

## Conclusion

In patients with intestinal failure on HPN the occurrence of low grade fewer, night sweats, declining performance status, low albumin and sometimes increased bilirubin usually prompts the diagnosis of a line related infection. This is because about 30% of line related infections in this cohort do not present with typical symptoms of high grade fever and rigors upon start of a new infusion but rather with those atypical symptoms [28]. In addition line related infections are the most frequent complication in HPN patients occurring with a frequency between two episodes per year and one episode every three years [28]. Nevertheless the diagnosis of line related infection could not be firmly established in either of these patients and empiric therapy was not successful. Instead tuberculous lymphadenitis was diagnosed and successfully treated. Predisposition for acquiring or reactivating tuberculosis may have been facilitated by the fact that patients with SBS receiving long term HPN have an impaired immune response [29]. Furthermore patient 1 was receiving steroids over a prolonged period of time system. It must also be noted that patient 2 may have had an atypical mycobacterial infection. On the other hand, tuberculosis, especially tuberculous lymphadenitis, has not been recognized as a specific problem in intestinal failure patients on HPN yet. So how may these conditions be related other than by chance?

Both patients carried a mutation in the NOD2 gene (patient 1: 1007 fs, patient 2: R702W). *NOD2* is an intracellular pattern recognition receptor which recognizes muramyl dipeptide (MDP) as part of peptidoglycan of the bacterial cell wall [30,31]. The cumulative incidence (homozygous, heterozygous and compound heterozygous) of mutations in the 3 major *NOD2* SNPs (R702W, G908R and 1007 fs) is 13.6% in a cohort of healthy controls [7]. Recently we and others have reported an increased frequency of NOD2 mutations in SBS patients [26,32]. At present it is not clear, whether a defect in NOD2 signaling leads to an altered response to operative stress ultimately resulting in a SBS or whether intestinal adaptation to the SBS situation is diminished resulting in long term HPN [26].

*M. tuberculosis* is mainly recognized via PRR like TLRs and NOD2. Certain mutations in the TLR genes, e.g. TLR1 and TLR6 are associated with an increased risk of acquiring *M. tuberculosis*[5,6]. In one study, host cells after exposure to *M. tuberculosis* were sensing the microbe-associated molecular pattern (MAMP) using independent PRRs like NOD2 and TLR. The study showed, that these receptors were non-redundant and interacted with each other synergistically [33]. In addition monocytes from patients homozygous for the 1007 fs mutation (3020insC) show diminished TNF and IL-10 cytokine response after stimulation with *M. tuberculosis* compared to heterozygous or homozygous wild-type controls. A cohort study in 377 African Americans with tuberculosis found that certain SNPs in the NOD2 gene were associated with either resistance or susceptibility to tuberculosis [34]. Nevertheless NOD2 mutations have not been firmly established as a risk factor for tuberculosis and several studies argue against such a correlation [35,36].

Thus, NOD2 mutations may be the common risk factor for both patients to develop intestinal failure requiring HPN and to acquire or reactivate tuberculosis, in these two cases tuberculous lymphadenitis. From a clinical point of view these two cases highlight the importance to search for alternative infectious complications other than line related blood stream infection in patients with SBS on HPN, including tuberculosis.

### Consent

Written informed consent was obtained from both patients for publication of this Case report and any accompanying images. A copy of the written consent from both patients is available for review by the Editor of this journal.

## Abbreviations

ESR: Erythrocyte sedimentation rate; FDG-PET: Fluordeoxyglucose positron emission tomography; GvHD: Graft-versus-host-disease; HPN: Home parenteral nutrition; IL: Interleukin; JAK2: Janus kinase 2; LDH: Lactate dehydrogenase; MAMP: Microbe-associated molecular pattern; NAAT: Nucleic Acid Amplification Test; NOD: Nucleotide-binding oligomerization domain receptors; PCR: Polymerase chain reaction; PRR: Pattern recognition receptor; SBS: Short bowel syndrome; SNP: Single nucleotide polymorphisms; TBC: Tuberculosis; TLR: Toll-like receptor; TNF: Tumor necrosis factor.

## Competing interests

All authors declare that they have no competing interests.

## Authors’ contributions

GL and HS gathered the information about the two patients. CH performed the mutational analysis. MT and SF collected the data from the FDG PET CT scans and analyzed them. JSF completed the microbiological part in Material and Methods. GL and HS wrote the manuscript. All authors read and approved the final manuscript.

## Pre-publication history

The pre-publication history for this paper can be accessed here:

http://www.biomedcentral.com/1471-230X/14/43/prepub
